# Endoplasmic Reticulum Malfunction in the Nervous System

**DOI:** 10.3389/fnins.2017.00220

**Published:** 2017-04-25

**Authors:** Joanna Jung, Marek Michalak, Luis B. Agellon

**Affiliations:** ^1^Department of Biochemistry, University of AlbertaEdmonton, AB, Canada; ^2^School of Dietetics and Human Nutrition, McGill UniversitySte. Anne de Bellevue, QC, Canada

**Keywords:** calnexin, proteostasis, endoplasmic reticulum, lipidostasis, neurological disorders

## Abstract

Neurodegenerative diseases often have multifactorial causes and are progressive diseases. Some are inherited while others are acquired, and both vary greatly in onset and severity. Impaired endoplasmic reticulum (ER) proteostasis, involving Ca^2+^ signaling, protein synthesis, processing, trafficking, and degradation, is now recognized as a key risk factor in the pathogenesis of neurological disorders. Lipidostasis involves lipid synthesis, quality control, membrane assembly as well as sequestration of excess lipids or degradation of damaged lipids. Proteostasis and lipidostasis are maintained by interconnected pathways within the cellular reticular network, which includes the ER and Ca^2+^ signaling. Importantly, lipidostasis is important in the maintenance of membranes and luminal environment that enable optimal protein processing. Accumulating evidence suggest that the loss of coordinate regulation of proteostasis and lipidostasis has a direct and negative impact on the health of the nervous system.

## Introduction

Neurodegenerative disorders are diseases of the nervous system, often chronic, and progressive in nature, affecting many people worldwide and increasing in incidence each year^1^. They account for about 1% of deaths worldwide and pose one of the largest health, economic, and social capital burden. Environmental factors such as lifestyle, diet, and stress are high risk factors for developing neurological disorders (Migliore and Coppedè, 2009; Ochoa-Repáraz and Kasper, 2014; Perry and Holmes, 2014; Rothhammer and Quintana, 2016). Impaired cellular homeostasis is a hallmark of neurodegenerative diseases (Hetz and Mollereau, 2014). The maintenance of cell homeostasis is a complex and dynamic process relying on coordinated functions of the cellular reticular network, the interconnected network of membranes within the cell that includes the endoplasmic reticulum (ER). The ER is a dynamic membrane system and a multifunctional organelle. It is a major site of protein and lipid synthesis (Hebert and Molinari, 2007; Schwarz and Blower, 2016), and the major intracellular store of Ca^2+^ that is used by Ca^2+^ signaling processes (Krebs et al., 2015). The purpose of this article is to discuss the dynamic events coordinated by the ER, namely synthesis, quality control, and degradation of proteins and lipids, sensing of cellular lipid status as well as maintenance of the ER Ca^2+^ in the cellular signaling network that influence cellular proteostasis and lipidostasis, in the context of the pathogenesis of the diseases of the nervous system.

## Cellular stress responses in the nervous system

**Figure 1:**
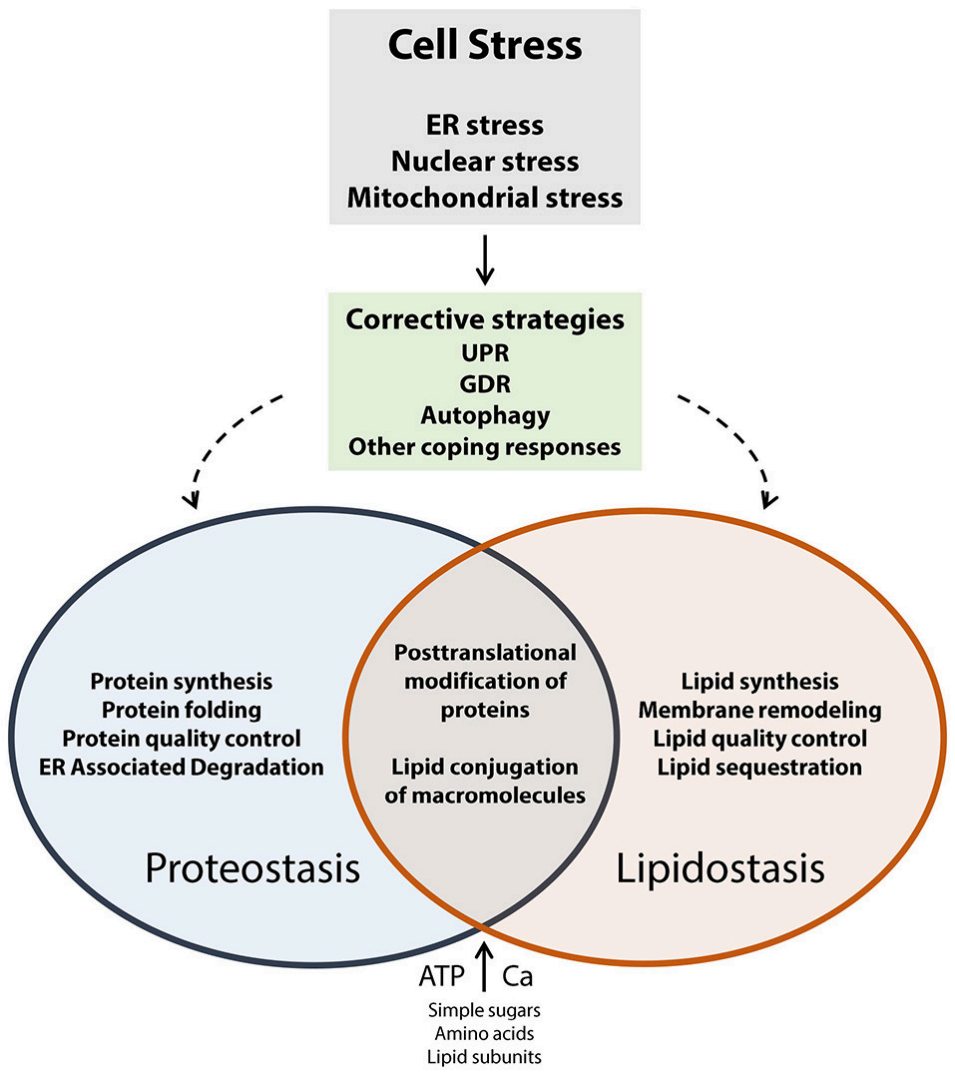
**Cell stress coping responses and the interplay between proteostasis and lipidostasis**. Proteostasis refers to optimal protein biosynthesis and trafficking whereas lipidostasis pertains to optimal lipid biosynthesis, trafficking, and membrane assembly. Both of these processes rely on the availability of energy (ATP), and nutrients (such as Ca^2+^, sugars, amino acids, lipid subunits, nucleotides, other essential cofactors). When cells experience external or internal insults that result in the loss of control of nutrient and energy metabolism corrective strategies (UPR, GDR, autophagy, other coping responses) are activated to counteract and eliminate cell stress. The regulatory and metabolic pathways that operate to recover proteostasis and lipidostasis are interconnected, and support each other in preserving global cellular homeostasis.

Cells, including neuronal cells (neurons, glial cells), are exposed to a wide variety of internal and external factors that induce cellular stress. These factors include gene variations that alter protein structure and function, inducers of oxidative stress, viral infection, environmental toxins, drugs, extremes in temperature, extremes in pH, inflammatory cytokines, lipotoxicity, Ca^2+^ depletion, aging, and other factors that cause loss of nutrient or energy homeostasis. Neurons are particularly susceptible to cellular stress, and disrupted cellular proteostasis or lipidostasis, due to their unique architecture and functional specialization (connectivity and excitability). In response to cellular stress, cells most frequently turn to the coping mechanisms such as the unfolded protein response (UPR; Groenendyk et al., 2013) and genome damage response (GDR; Dicks et al., 2015; Figure 1). The UPR works to restore protein homeostasis in the ER (Groenendyk et al., 2013; Hetz and Mollereau, 2014) whereas the GDR functions to repair DNA or chromatin damage (Dicks et al., 2015). Several recent review articles discuss these topics in greater depth (Cao and Kaufman, 2013; Groenendyk et al., 2013; Hetz and Mollereau, 2014; Wang and Kaufman, 2014; Dicks et al., 2015; Hetz et al., 2015). Disrupted proteostasis has been identified as an underlying cause of many neurodegenerative diseases including Alzheimer's disease, Parkinson's disease, Huntington disease, amyotrophic lateral sclerosis, prion related diseases, all of which have been referred to as diseases of protein folding (Hetz and Mollereau, 2014). These examples illustrate that long term alteration of cellular function in response to chronic disruption of proteostasis in the nervous system eventually lead to the pathogenesis of neurodegenerative disorders.

## Lipid homeostasis and neurodegenerative diseases

The ER is a critical organelle for maintenance of cellular lipid homeostasis (van Meer et al., 2008). It is the site of synthesis of the bulk of structural phospholipids, sterols, and storage lipids such as triacylglycerols and sterol esters (Higgins, 1974; Ikonen, 2008; Fagone and Jackowski, 2009; Chauhan et al., 2016). This organelle also supplies lipids to other cellular organelles, and is the driver of cellular lipid homeostasis. The brain is the most cholesterol enriched organ in the body (Dietschy and Turley, 2001; Zhang and Liu, 2015). Cholesterol in brain cells is derived primarily from *de novo* synthesis since lipoproteins are unable to cross the blood-brain barrier (BBB; Valdez et al., 2010; Zhang and Liu, 2015; Mistry et al., 2017). The majority of the cholesterol in the brain is found in the myelin sheaths that surround axons.

Impaired metabolism and transport of lipids in the brain has been linked to many neurodegenerative diseases such as Alzheimer's disease, Huntington disease, Parkinson's disease, multiple sclerosis, amyotrophic lateral sclerosis, including inherited neurological diseases such as Niemann-Pick C disease, Smith-Lemli-Opitz syndrome, and Gaucher's disease (Cutler et al., 2002; Vanier, 2010; Wu G. et al., 2011; Don et al., 2014; Petrov et al., 2016; Schultz et al., 2016; Abdel-Khalik et al., 2017; Kim et al., 2017; Mistry et al., 2017; Schuchman and Desnick, 2017). In the case of amyotrophic lateral sclerosis, accumulation of ceramides, and cholesteryl esters which cause death of motor neurons (Cutler et al., 2002) is associated with defects in the metabolism of sterols (Cutler et al., 2002; Abdel-Khalik et al., 2017). Lipids may also affect the function of certain proteins; for example, the degree of membrane insertion of huntingtin, the brain protein involved in Huntington disease, is influenced by the amount of membrane cholesterol (Gao et al., 2016).

It is probable that inappropriate remodeling of membranes potentiates the loss of proteostasis by causing the malfunction of molecular chaperones and other membrane bound proteins (Figure 1). A recent study reported that long term feeding of mice with a diet enriched with saturated fats causes significant remodeling of the brain lipidome, particularly those lipids that make up the cell membrane (Giles et al., 2016). Considering the integral role of the ER in lipid synthesis, transport and degradation, we propose that lipidostasis is an emerging and significant risk factor in the pathogenesis of neurodegenerative diseases.

## Proteostasis and calnexin

The ER protein quality control system is comprised of many molecular chaperones and folding enzymes that closely monitor and facilitate the folding of proteins and their secretion in order to prevent formation and accumulation of toxic protein aggregates. Calnexin, calreticulin, and PDIA3 (a protein foldase that catalyzes the formation and correct isomerization of disulfide bonds and interacts with both calnexin and calreticulin), are the core components of the ER protein quality control system (Hebert and Molinari, 2007). Folding of most of non-glycosylated proteins is supported by BiP/GRP78, a protein that interacts with hydrophobic regions of newly synthesized proteins (Hebert and Molinari, 2007; Halperin et al., 2014). Other chaperons including GRP94, ERdj3, cyclophilin B, PDI, PDIA4, SDF2, and additional members of the PDI family proteins form large protein folding complexes that interact with misfolded and unfolded proteins (Hebert and Molinari, 2007; Halperin et al., 2014) to assist in their proper processing. Moreover, a class of small molecules, termed proteostasis promoters (Vega et al., 2016), have been identified.

Calnexin is a type I transmembrane molecular chaperone, and is of special interest as this protein is highly expressed during the development of the nervous system (Coe et al., 2008; Kraus et al., 2010). In mice, calnexin deficiency causes dysmyelination of peripheral and central nervous system (PNS; Kraus et al., 2010; Jung et al., 2011) as a result of misfolding of P0 and PMP22, two essential glycoproteins required for myelin formation (Jung et al., 2011). Calnexin has also been shown to interact with myelin oligodendrocyte glycoprotein (MOG; Jung and Michalak, 2011; Jung et al., 2015), a protein that is critically involved in the myelination of nerve cells in the central nervous system (CNS). Although MOG is only a minor component of CNS myelin it plays an important role in the pathology of multiple sclerosis (MS), a progressive neurological disorder caused by an autoimmune response against antigens of the CNS. Autoantibodies against MOG have been detected in the serum of MS patients (Reindl et al., 2013). Although deficiency in calnexin does not impact on the intracellular trafficking of MOG, the folding and stability of MOG are affected (Jung and Michalak, 2011; Jung et al., 2015). The discovery of a role for calnexin in maintaining myelin sheets (Kraus et al., 2010; Jung et al., 2011) and folding of MOG (Jung et al., 2015) provides new and unanticipated insights into the mechanisms responsible for myelin diseases of the PNS and CNS.

Calnexin interacts with the SH3-domain GRB2-like (endophilin) interacting protein 1 (SGIP1), a neuronal regulator of endocytosis, supporting a role for calnexin in the recycling of synaptic membrane proteins and maintaining synaptic homeostasis (Li et al., 2011). The balance between exocytosis and endocytosis is vital in maintaining the function of the brain cells (Lim and Yue, 2015). Endocytosis might also be a potential mechanism involved in cell-to-cell transmission of protein aggregates that underlie the pathogenesis of neurodegenerative diseases stemming from accumulation of protein aggregates (Lim and Yue, 2015). Synaptic transporters such as the serotonin transporter (Tate et al., 1999) and glycine transporter 2 which are expressed in the CNS (Arribas-González et al., 2013) are also calnexin substrates. The appearance of calnexin on the surface of hippocampal neurons has been reported (Itakura et al., 2013), further supporting the participation of calnexin in the integration of synaptic proteins to the plasma membrane as well as in the maintenance of synaptic proteostasis.

Global knockout of the PDIA3 gene in mice is embryonic lethal (Coe et al., 2010), however, targeted knockout of PDIA3 in the murine nervous system leads to severe motor dysfunction and growth retardation associated with a loss of neuromuscular synapses reminiscent of calnexin deficiency (Kraus et al., 2010), and more recently, of amyotrophic lateral sclerosis in humans (Woehlbier et al., 2016). Association between PDIA3 and the amyotrophic lateral sclerosis may not be surprising as PDIA3 expression is high in the brain during embryonic development (Coe et al., 2010). BiP, a key component of the UPR and essential regulator of ER proteostasis and Ca^2+^ homeostasis, has also been associated with neurodegenerative diseases (Hoozemans et al., 2005; Carnemolla et al., 2009; Wang et al., 2009; Gorbatyuk and Gorbatyuk, 2013). Global BiP gene knockout in mice is embryonic lethal (Luo et al., 2006). However, targeted deletion of BiP in developing Schwann cells manifests in a phenotype reminiscent of that seen in calnexin-deficient mice (Kraus et al., 2010), in particular PNS myelin abnormalities, diminished number of myelinating Schwann cells and hind limb paralysis (Hussien et al., 2015; Volpi et al., 2016). A class of small molecules, termed proteostasis promoters (Vega et al., 2016), have been described. For example, valproic acid, a drug that is currently used in the clinical management of mood disorders (Chiu et al., 2013), has been shown to induce the UPR coping mechanism and inhibit ER stress (Kakiuchi et al., 2003; Lee et al., 2014; Wang et al., 2015; Peng et al., 2016). Although the precise mechanism of action of specific compounds are not yet fully deciphered, proteostasis promoters have in common the ability of enhancing protein processing and relieving cellular stress, including in neuronal cells.

Disrupted autophagy has been linked with pathology of CNS disorders (Nikoletopoulou et al., 2015). Autophagy, a dynamic process promoting self-digestion, to help eliminate toxic aggregates through the lysosomal pathway (Yorimitsu et al., 2006) involves bulk degradation of proteins, lipids and organelles, including the ER (Kaur and Debnath, 2015). As neurons are post-mitotic cells, they rely on autophagy for removal of defective organelles, protection against protein aggregation and in preventing the accumulation of toxic proteins. Abnormal autophagy is involved in neurodegenerative disease pathology (Nikoletopoulou et al., 2015) as well as in acute brain injuries (Galluzzi et al., 2016). Calnexin is a component of the early autophagosomes (Gagnon et al., 2002) pointing to its potential role in an alternative mechanism for degradation of misfolded proteins and removal of organellar membranes in the nervous system. The accumulating evidence from animal and clinical studies support a role for calnexin, and likely other ER molecular chaperones and folding enzymes, in maintaining neuronal proteostasis and perhaps also lipidostasis.

## ER calcium homeostasis

The ER is the major Ca^2+^ storage depot of the cell. Ca^2+^ release from the ER impacts on the vast majority of cellular processes, including cell proliferation, transcription, exocytosis, apoptosis (Corbett and Michalak, 2000; Prins and Michalak, 2011; Krebs et al., 2015). Accordingly, maintenance of normal ER Ca^2+^ capacity is vital in supporting cellular stress coping responses in re-establishing proteostasis and lipidostasis (Figure 1), and therefore ER Ca^2+^ levels must be finely regulated. This can be accomplished by coordinating the function of multiple Ca^2+^ sensors, pumps, channels, exchangers, and Ca^2+^ binding proteins (Prins and Michalak, 2011; Brini et al., 2014; Krebs et al., 2015). Ca^2+^ in the lumen of the ER is frequently depleted by Ca^2+^ signaling events occurring within the ER and in other cellular compartments. Thus, in order to maintain Ca^2+^ signaling capacity, Ca^2+^ released from the ER lumen must be replenished. This process, which involves Ca^2+^ entry from the external environment of the cell into the ER, is referred to as store-operated Ca^2+^ entry (SOCE; Soboloff et al., 2012).

SOCE is initiated by Ca^2+^ release through inositol 1,4,5-triphosphate receptor (IP_3_R) and/or ryanodine receptor Ca^2+^ channels and relies on ER luminal Ca^2+^ sensors (STIM proteins), a plasma membrane Ca^2+^ channel (ORAI), and sarco-endoplasmic reticulum Ca^2+^-ATPase (SERCA; Soboloff et al., 2012). Since ER chaperones and folding enzymes require Ca^2+^ to function, the sustained depletion of ER Ca^2+^ leads to the accumulation of misfolded proteins which subsequently activates UPR and other corrective strategies (Groenendyk et al., 2013). In neuronal tissue, Ca^2+^ signaling is especially important as it controls additional processes that do not occur in other tissues, such as synaptic signaling and neurotransmission. Neuronal Ca^2+^ signaling also plays an important role in learning, memory and neuronal plasticity (Brini et al., 2014). Not surprisingly, disturbance of ER Ca^2+^ homeostasis is commonly observed in severe neurodegenerative diseases (Mattson et al., 2000; Ong et al., 2010; Chen et al., 2011; Mekahli et al., 2011; Wu J. et al., 2011; Belal et al., 2012; Selvaraj et al., 2012; Bezprozvanny and Hiesinger, 2013; Popugaeva and Bezprozvanny, 2013; Zeiger et al., 2013; Koran et al., 2014). For example, mutations in the IP3R type 1 gene leads cerebellar neurodegeneration in mice and causes spinocerebellar ataxia type 15 (SCA15) leading to neurodegeneration in humans (van de Leemput et al., 2007; Sasaki et al., 2015; Tada et al., 2016). Mechanisms that ensure ER Ca^2+^ homeostasis might allow neuronal cells to effectively maintain both proteostasis and lipidostasis, and thereby prevent neuronal pathology. Overload of Ca^2+^ in the ER is also harmful hence ensuring constant supply without regulated release could lead to disease. Increased abundance of STIM1 and ORAI1 in HEK cells resulted in reduced formation and secretion of Aβ peptides (Zeiger et al., 2013). Furthermore, neuronal cell expressing mutant Huntingtin protein exhibit enhanced SOCE (Wu J. et al., 2011) and the loss of SOCE was observed in neuroblastoma cells treated with agent that mimics Parkinson's disease in mice (Selvaraj et al., 2012). Mechanisms that ensure the constant supply of Ca^2+^ in the ER might allow neuronal cells to effectively maintain both proteostasis and lipidostasis, and thereby prevent neuronal pathology.

## Brain permeability

The BBB is a physical structure that separates the CNS from the rest of the body, and selectively controls the flow of molecules in and out of the brain. Dysfunction of the brain endothelial cells, essential component of the BBB, is involved in the pathology of many CNS disorders (Deane et al., 2004; Cirrito et al., 2005; Zlokovic et al., 2005; Alvarez and Teale, 2006; Deane and Zlokovic, 2007; Tietz and Engelhardt, 2015), however the molecular mechanisms underlying its contribution are not fully understood. Abnormalities in BBB have been linked to pathogenesis of the Alzheimer disease (Cirrito et al., 2005; Zlokovic et al., 2005) involving defective clearance of β-amyloid (Deane et al., 2004; Deane and Zlokovic, 2007). Recent studies link ER stress coping responses and BBB disruption in the rat model of epilepsy (Ko et al., 2015). Brain endothelial cells are not only a physical barrier but also a dynamic interface involved in transport of the molecules and capable of response to inflammation on either side of the barrier. Brain endothelial cells are sensitive to proinflammatory factors, which affects the integrity and function of the BBB, originating from both sides of the BBB (Tietz and Engelhardt, 2015). The crossing of the auto-reactive lymphocytes across BBB accompanied by demyelination and neurodegeneration are hallmarks of MS pathology (Mahad et al., 2015). Experimental autoimmune encephalomyelitis (EAE), an animal model of MS allowed insights into a potential role of ER chaperones in initiation and progression of MS. ER quality control components including calnexin, calreticulin, BiP and PDIs likely play critical roles in facilitating the folding and trafficking of endothelial specific proteins such as ICAM, VCAM, and p-selectin in response to inflammation. Increased abundance of BiP has been seen in brain of MS patients (Mháille et al., 2008; Cunnea et al., 2011) and conditional knockout of the BiP gene and, consequently a disrupted proteostasis, exhibits exacerbated EAE symptoms that are not related to altered inflammatory response (Hussien et al., 2015). It is conceivable that other components of protein quality control, including PDIA3, calreticulin, and calnexin, may influence the function and integrity of the BBB. For example, calreticulin associates with MMP9 (Duellman et al., 2015) a matrix metalloproteinase that is critical for the integrity of BBB (Dubois et al., 1999; Rosenberg, 2009) and contributes to amyloid formation and clearance (Nalivaeva et al., 2008). Strategies allowing exogenous manipulation of the ER protein quality control system may offer a means to regain proteostasis as well as lipidostasis (Figure 1) in the nervous system, and assist in the management of neurological disorders.

## Summary

We propose that disrupted proteostasis and lipidostasis underlie many neurological disorders. Recent studies suggest that molecular chaperones are intimately involved in coordinating the cellular proteostasis and lipidostasis in the nervous system, including the cells that make up the BBB, by ensuring the quality of key proteins and lipid components of the membranes. Importantly, the activity of ER chaperones depends on ER Ca^2+^ homeostasis. A detailed knowledge of the regulatory and metabolic pathways involved in proteostasis and lipidostasis in cells that make up the nervous system, will provide better insights into the heterogeneity of neurological disorders and uncover new opportunities for therapeutic development.

## Author contributions

All authors listed, have made substantial, direct and intellectual contribution to the work, and approved it for publication.

## Funding

This work was supported by Canadian Institutes of Health Research grants MOP-15291, MOP-15415, MOP-53050 to MM; MOP-15291 and MOP-86750 to LA; and by a Natural Sciences and Engineering Research Council of Canada Discovery grant to LA.

### Conflict of interest statement

The authors declare that the research was conducted in the absence of any commercial or financial relationships that could be construed as a potential conflict of interest.

## Footnotes

^1^http://www.who.int/mental_health/publications/neurological_disorders_ph_challenges/en/
